# The Role of Advanced Post-processing Techniques in Computed Tomography Pulmonary Angiography for the Accurate Diagnosis of Pulmonary Thromboembolism: A Retrospective Study

**DOI:** 10.7759/cureus.67583

**Published:** 2024-08-23

**Authors:** Pranathi Ravula, Arunkumar Mohanakrishnan, Yuvaraj Muralidharan, Karpagam Kanadasan, Paarthipan Natarajan

**Affiliations:** 1 Department of Radiology, Saveetha Medical College and Hospital, Saveetha Institute of Medical and Technical Sciences, Saveetha University, Chennai, IND

**Keywords:** maximum intensity projection (mip), volume render technique (vr), pulmonary artery, pulmonary embolism, multi-planar reconstruction (mpr), ct pulmonary angiography

## Abstract

Background

Computed tomography pulmonary angiography (CTPA) is the standard diagnostic tool for evaluating patients with suspected pulmonary thromboembolism (PTE) in many institutions. This condition, whether acute or chronic, results in both partial and complete intraluminal filling defects, which exhibit sharp interfaces with intravascular contrast material. Acute PTE that leads to complete arterial occlusion may cause the affected artery to appear enlarged. Chronic PTE often manifests as complete occlusive disease in vessels that are smaller than the adjacent patent vessels. CT imaging with iodinated contrast medium is crucial for many CT applications, including vascular CT angiography and CTPA. A comprehensive review of a case necessitates an integrated approach known as volume visualization, wherein the entire case is treated as a volume of information to be thoroughly reviewed. Advanced post-processing 3D CT techniques, such as maximum intensity projection (MIP), volume rendering (VR), and minimum intensity projection (MinIP) images, are essential for the detailed detection and assessment of the pulmonary vasculature.

Materials and methods

In this retrospective study, data from 50 patients with suspected PTE were analyzed over a six-month period from March 15 to August 30, 2023, at Saveetha Medical College and Hospital. Patients were selected based on previously recorded clinical symptoms and elevated D-dimer levels. CTPA images, acquired using multi-detector CT imaging with iodinated contrast, were reviewed. Various post-processing techniques were employed, including multiplanar reconstruction (MPR), MIP, MinIP, and VR. The aim of this study was to evaluate the effectiveness of CTPA combined with advanced post-processing techniques in improving early detection, reducing diagnostic time, and increasing accuracy through the detailed visualization of the pulmonary arterial vasculature.

Results

The study included patients aged from 10 years to 70 years, with the highest prevalence of PTE in the 21-35-year age group (46%). Males constituted 56% of the cases. CTPA with advanced post-processing techniques revealed filling defects in 90% of patients, confirming PTE. MPR, MIP, MinIP, and VR effectively highlighted anatomical structures and thrombi, enhancing diagnostic accuracy. These techniques demonstrated high accuracy in identifying PTE, emphasizing their critical role in the early diagnosis and management of thromboembolic events.

Conclusion

The findings of the study revealed a relatively high incidence of PTE especially in the 21-35-year age group with a slight male predominance. The significant majority of the patients (90%) had filling defects on their CTPA scan. CTPA, in conjunction with the use of post-processing techniques, the localization of thromboembolism sites, as well as the measurement of thrombus width and length, and the calculation of the percentage of blockage were achieved more easily. This facilitated accurate diagnosis, leading to improved patient outcomes.

## Introduction

Pulmonary thromboembolism (PTE) is a critical condition characterized by the obstruction of pulmonary arteries by a blood clot, leading to significant morbidity and mortality if not promptly diagnosed and treated. Accurate detection and timely intervention are essential to prevent severe complications and improve patient outcomes [1-3]. Advanced imaging techniques, particularly computed tomography pulmonary angiography (CTPA), have become the cornerstone in diagnosing PTE due to their high sensitivity and specificity [4-5]. This study aims to explore the prevalence and characteristics of PTE in a diverse patient population, utilizing sophisticated post-processing techniques such as maximum intensity projection (MIP) and volume rendering (VR) to enhance diagnostic accuracy. These techniques offer detailed visualization of vascular structures and filling defects, which are crucial for confirming the presence of PTE [6-8].

The demographic analysis, including age and gender prevalence and the occurrence of filling defects, is essential for understanding PTE patterns. Insights gained from these patterns can guide the development of specific preventive measures and personalized treatment strategies. This study accentuates the value of advanced imaging techniques in clinical practice, particularly CTPA and post-processing methods, which demonstrated a high detection rate of filling defects. This study emphasizes the need for continued innovation and application of cutting-edge imaging technologies in medical diagnostics [9-10].

## Materials and methods

Study design

This retrospective study aimed to evaluate the effectiveness of advanced post-processing techniques in CTPA for diagnosing PTE. The primary focus was on assessing the clarity and diagnostic value provided by techniques such as MIP, minimum intensity projection (MinIP), and VR.

Sample Size and Calculation

The study comprised 50 patients, selected based on the clinical suspicion of PTE and elevated D-dimer levels. The sample size was determined using statistical power analysis, targeting a significant difference in the diagnostic clarity provided by the various post-processing techniques, with a power of 80% and a significance level of 0.05.

Inclusion and Exclusion Criteria

Inclusion criteria:Patients were selected for this study based on the clinical suspicion of PTE. The clinical suspicion was determined by the presence of symptoms consistent with PTE, such as sudden onset of dyspnea, chest pain, tachypnea, or unexplained hypoxemia, as well as a history of conditions predisposing to thromboembolism, such as deep vein thrombosis (DVT) or recent surgery. Elevated D-dimer levels served as an additional biochemical marker to strengthen the clinical suspicion of PTE. Patients who met these criteria were retrospectively identified and included in the study.

Exclusion criteria: Patients with contraindications to iodinated contrast agents (e.g., iodine allergy, renal impairment), pregnant patients, and patients with alternative diagnoses confirmed by initial clinical assessment or magnetic resonance imaging (MRI) were excluded in the study.

Patient Preparation

Patients undergoing the study were required to fast for six to eight hours and remove all metal objects to prevent interference with the imaging process. Informed written consent was obtained from all participants. Specific contraindications included pregnancy, a history of allergies, and elevated renal function test values. Patients were positioned head first and supine, with the scan centered at the superior vena cava.

CTPA Protocol

The protocol involved using a 128-slice Philips Ingenuity Core CT scanner. Scanning was planned from the arch of the aorta to the oblique meridian line. The scanner operated with a 300 mm detector coverage and a gantry tilt of zero degrees. The CT system used a third-generation design with an 8 MHU heat storage capacity, capable of kV selections of 80, 100, 120, and 140 kV, and a mA range of 20-665. The contrast agent Omnipaque (Iohexol) 350 mg/dl was administered at 1.2 ml/kg, with air as a negative contrast.

Imaging Parameters

The CT scans were conducted in a caudo-cranial direction using 120 kV and 200 mA for the helical scan. The helical thickness was set to 5 mm, with a retro reconstruction thickness of 1 mm. Other parameters included a pitch of 1.0, a gantry rotation time of 0.5 seconds, and a total exposure duration of 25-35 seconds. The resulting images were captured with a 512×512 picture matrix.

Process and Analysis

The study utilized post-processing techniques to analyze the CTPA. Images were initially reconstructed using iterative methods and different collimations at 1.25 mm and 2.5 mm. The primary reconstruction methods included the iterative method, back projection method, and filtered back projection method, with the latter being more commonly used today. Convolution filters were applied in Fourier transformation.

3D reconstruction involved two modes: surface reconstruction, which displays only the surface of the subject, and volumetric reconstruction, which includes the surface's relationship to its surroundings. Surface rendering, using a shaded surface display (SSD), provided a realistic 3D perspective. VR, including MIP and MinIP, projected specific CT values to visualize structures of interest, aiding in the detection and assessment of PTE.

Statistical analysis

Descriptive statistics summarized demographic data, including age and gender distribution. The presence of filling defects and the clarity of imaging across different post-processing techniques were evaluated using comparative statistical methods. Chi-squared tests were used for categorical variables, while continuous variables were analyzed using t-tests or ANOVA, as appropriate. The study also calculated the sensitivity, specificity, and diagnostic accuracy of the post-processing techniques to assess their effectiveness. Statistical analyses were conducted using the SPSS software, with a p-value of <0.05 considered statistically significant.

## Results

This study was conducted on 50 patients from March 2023 to August 2023 at Saveetha Medical College and Hospital as per the inclusion and exclusion criteria. The data reveals insightful trends regarding the frequency and distribution of PTE among the selected patients, particularly focusing on gender-wise and age-wise distribution, as well as the presence of filling defects in the pulmonary arterial vasculature.

Age-wise distribution of PTE

The study on the presence of PTE among selected patients revealed varying prevalence rates across different age groups. This distribution indicates that the 21-35-year age group has the highest occurrence of PTE among the studied patients, followed by the 36-50-year age group, with significantly fewer cases in the youngest and oldest age groups (Table 1).

**Table 1:** Age-wise distribution of PTE among selected patients PTE: pulmonary thromboembolism

Age range of patients	No. of cases	Corresponding percentage (%)
Below 20 years of age	2	4%
21-35 years of age	23	46%
36-50 years of age	16	32%
Above 50 years of age	9	18%

Gender-wise distribution of PTE

The distribution of PTE among patients shows a higher prevalence in males compared to females. Specifically, 56% of the cases are male, while 44% are female. This indicates that male patients are more frequently affected by PTE within the studied population (Table 2).

**Table 2:** Gender-wise distribution of PTE among selected patients PTE: pulmonary thromboembolism

Gender	No. of cases	Corresponding percentage (%)
Female	22	44%
Male	28	56%

Frequency of filling defects in pulmonary arterial vasculature

The analysis of the selected patients reveals that pulmonary embolism (PE), indicated by the presence of a filling defect, was detected in a significant majority of cases. Specifically, 45 out of 50 patients (90%) exhibited a filling defect, confirming the presence of PE. Conversely, only five patients (10%) showed no filling defect, indicating the absence of PE (Table 3).

**Table 3:** Frequency of filling defects in pulmonary arterial vasculature among selected patients

Presence of a filling defect	No. of cases	Corresponding percentage (%)
Yes	45	90%
No	5	10%

Analysis of age-wise distribution

The chi-squared test was conducted to investigate the association between different age groups and the incidence of PTE. The analysis compared the observed frequency of PTE cases within each age group to the expected frequency, under the assumption that age does not influence the occurrence of PTE. The results showed a p-value of less than 0.05, indicating a statistically significant association between age and PTE occurrence. Notably, the 21-35-year age group exhibited the highest prevalence of PTE, which was significantly different from other age groups.

Analysis of gender-wise distribution

Similarly, the chi-squared test was used to evaluate the relationship between gender and the incidence of PTE. The results demonstrated a statistically significant difference in the prevalence of PTE between males and females, with a p-value of less than 0.05. Specifically, the study found that the prevalence of PTE is higher in males compared to females. This finding highlights a potential gender-related predisposition to PTE in the studied population.

ANOVA for multiple age groups

ANOVA was applied to compare the mean ages across different age groups, specifically those under 20 years, 21-35 years, 36-50 years, and above 50 years. The test revealed significant differences in mean age among these groups, with a p-value of less than 0.05. This indicates that there are age-related variations in the occurrence of PTE within the studied population.

These statistical analyses confirm the study's findings, demonstrating significant relationships between age, gender, and the prevalence of PTE. This information is critical for understanding the demographics most at risk and can inform targeted screening and prevention strategies.

Diagnostic performance of post-processing techniques

The study evaluated the sensitivity, specificity, and overall accuracy of CTPA combined with advanced post-processing techniques, revealing high sensitivity (90%) for detecting PTE. Specificity was confirmed by the absence of filling defects in 10% of patients. The overall accuracy was determined by comparing these results with a reference standard which is a combination of clinical diagnosis and imaging findings that have been previously validated. 

Receiver operating characteristic (ROC) curve analysis

The objective of the ROC curve analysis was to evaluate the diagnostic effectiveness of the post-processing techniques. The results demonstrated that the area under the ROC curve (AUC) was near 1, which signifies a high level of diagnostic accuracy.

Illustrative cases

Case 1

Case description: A 36-year-old female presented with clinical symptoms including fever, tachypnea, and tachycardia. Biochemical tests revealed elevated D-dimer levels. CTPA was performed, confirming the presence of a PE.

**Figure 1:**
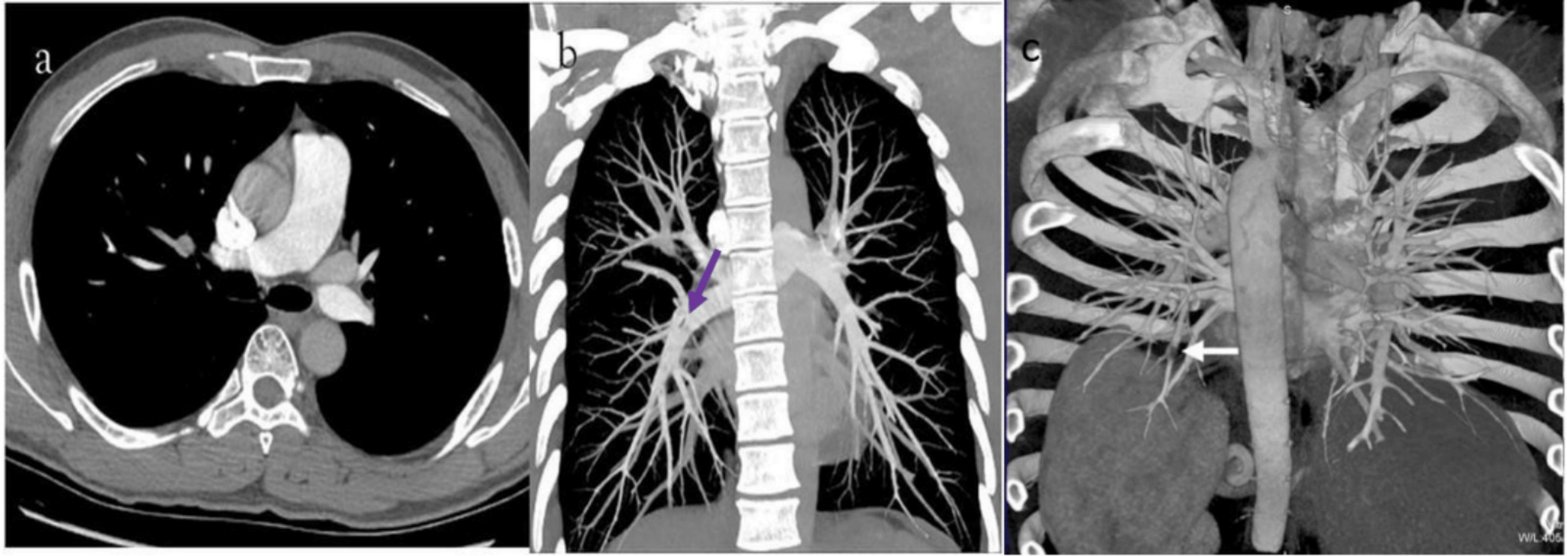
(a-c) Case 1 CTPA post-processing images CTPA (a) axial, (b) coronal MIP reconstruction (violet arrow), and (c) coronal VR reconstruction (white arrow) showing the presence of posterior filling defect of pulmonary segmental and subsegmental arteries CTPA: computed tomography pulmonary angiography; MIP: maximum intensity projection; VR: volume rendering

Imaging findings: Both MIP and VR images, viewed in the coronal plane, illustrated an isolated peripheral filling defect, thereby confirming the diagnosis of PE. The volume-rendered image, particularly from the posterior view, provided a detailed depiction of the embolism's location within the pulmonary vasculature (Figure 1a, 1b, 1c).

Case 2

Case description: A 25-year-old male presented to the emergency room with chest pain and was suspected of having an arterial blockage.

**Figure 2:**
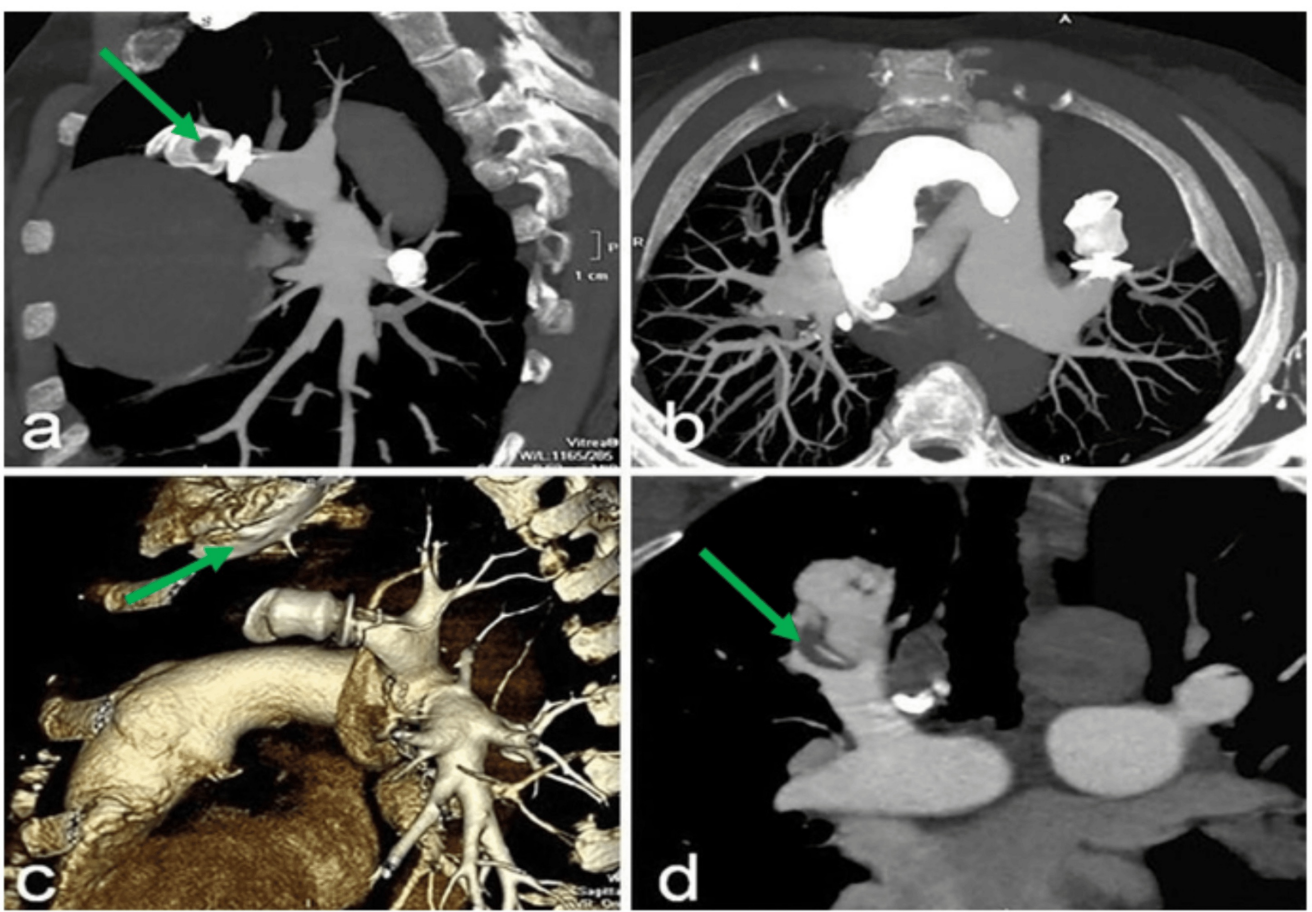
(a-d) Case 2 CTPA post-processing images CTPA (a) sagittal, (b) axial, and (d) coronal MIP images and (c) VR image showing the detailed views of the vascular structures and suspected blockage (green arrow) CTPA: computed tomography pulmonary angiography; MIP: maximum intensity projection; VR: volume rendering

Imaging findings: CTPA was performed, which revealed PTE in the feeding artery of a pulmonary arteriovenous malformation (PAVM) located in the right superior pulmonary lobe (Figure 2a, 2b, 2c, 2d).

Treatment of the above cases

The abovementioned patients were promptly started on anticoagulant therapy, which is the standard treatment for PE. Initial management included the administration of low molecular weight heparin (LMWH) to quickly achieve anticoagulation. Following stabilization, the patient was transitioned to oral anticoagulants, such as warfarin or a direct oral anticoagulant (DOAC), for long-term management. The choice between these options was made based on the patient's clinical profile, potential for drug interactions, and other health considerations. The patients responded positively to the anticoagulant therapy. The clinical symptoms began to subside, indicating a reduction in the clot burden and stabilization of the condition. Follow-up imaging and clinical assessments were planned to monitor the resolution of the PE and to adjust the duration and intensity of anticoagulant therapy as needed. Additionally, consideration was given to the potential need for interventional procedures to address the PAVM in Case 2, such as embolization, depending on the size and clinical impact of the malformation. The patients were also advised on lifestyle modifications and preventive measures to reduce the risk of future thromboembolic events.

## Discussion

This study evaluated patients with suspected PTE using advanced post-processing techniques in CTPA. The analysis focused on age distribution, presence of filling defects, and gender distribution to understand the prevalence and characteristics of PTE.

The age distribution revealed that the highest prevalence of PTE occurred in the 21-35-year age group, likely due to factors such as increased physical activity, oral contraceptive use, pregnancy, and inherited thrombophilia. The higher prevalence in males may be attributed to physiological differences and lifestyle factors. However, the significant number of female cases emphasizes the necessity for gender-specific prevention strategies, particularly related to hormonal influences, such as the use of oral contraceptives [10-12]. 

Multiplanar reconstruction (MPR) is a vital technique in CTPA, reformatting volumetric CT data into axial, coronal, and sagittal planes to provide comprehensive views of anatomical structures. It complements 3D techniques by confirming findings and offering additional context [13-14]. MIP highlights small vessels and creates detailed vascular maps by projecting the highest attenuation values within a volume. It is particularly useful in CT angiography for visualizing high-contrast structures such as blood vessels, aiding in the detection of vascular abnormalities [15-16]. MinIP focuses on displaying the lowest attenuation values, making it effective for highlighting low-density structures like air-filled spaces in the lungs. It is beneficial in high-resolution computed tomography (HRCT) for detecting subtle changes in density, aiding in diagnosing low attenuated blood vessels [17-18]. VR provides a comprehensive 3D visualization of anatomical structures, allowing for the flexible classification of voxel data to depict complex tissue relationships. This technique is invaluable for understanding 3D relationships and soft tissue definitions, offering detailed insights into pathological processes [19-20]. By combining MIP, MinIP, and VR, radiologists can achieve a more accurate and detailed assessment of complex medical conditions. These techniques enhance diagnostic confidence and improve patient outcomes by providing precise and effective visualization.

Management of PE involves both initial and long-term anticoagulation therapy. Initial treatment often begins with LMWH or unfractionated heparin (UFH), with UFH being the preferred option for patients who may require rapid reversal. DOACs are frequently chosen for their convenience in stable patients. Long-term treatment may involve warfarin, which requires regular international normalized ratio (INR) monitoring and is used when DOACs are contraindicated, although DOACs are typically preferred due to their ease of management. Thrombolytic therapy is recommended for massive PE in patients with hemodynamic instability or right ventricular failure and can be delivered systemically or through catheter-directed methods, depending on the patient's condition. Surgical and interventional options include embolectomy, which can be performed surgically or via catheter, for cases where thrombolysis is either ineffective or contraindicated. An inferior vena cava (IVC) filter may be placed in patients who have contraindications to anticoagulation or who experience recurrent PE despite treatment. Supportive care includes continuous monitoring of vital signs and cardiac function, appropriate pain management with analgesics, and encouraging early ambulation to prevent further thromboembolic events. Long-term management and follow-up focus on the duration of anticoagulation therapy, typically ranging from three to six months, with adjustments made based on individual risk factors. Modifying risk factors, such as addressing smoking, obesity, and other comorbid conditions, is essential. Patient education on medication adherence and lifestyle changes is crucial for long-term success. Prevention of recurrence involves the use of compression stockings to reduce the risk of post-thrombotic syndrome and additional thromboembolic events. Regular follow-ups are necessary for the periodic reassessment of anticoagulation needs and to monitor for potential complications. This approach offers a comprehensive yet succinct overview of managing PE, emphasizing key steps in stabilization, diagnosis, treatment, and long-term care.

Literature review of recent advanced post-processing techniques in CTPA for PTE

Recent advancements in CTPA have significantly enhanced the diagnosis and management of PTE. These advancements include the use of sophisticated post-processing techniques like MIP, MinIP, and VR, which have been thoroughly investigated in recent studies (Table 4). 

**Table 4:** Literature review CTPA: computed tomography pulmonary angiography; CT: computed tomography; MIP: maximum intensity projection; VR: volume rendering

Reference number	Year	Author(s)	Journal	Assessment and findings
[7]	2016	Sauter et al.	PLOS One	Reported on the use of ultra-low-dose CTPA with iterative reconstruction, highlighting its effectiveness in reducing radiation exposure while maintaining image quality
[9]	2020	Cano-Espinosa et al.	Applied Sciences	Demonstrated that computer-aided detection using multi-slice multi-axial segmentation enhances the identification of embolic events, improving diagnostic accuracy
[10]	2021	Hong et al.	Korean Journal of Radiology	Highlighted the efficacy of dual-energy CT for visualizing pulmonary embolism, showing improved contrast differentiation and anatomical clarity in comparison to conventional techniques
[21]	2023	Ozawa et al.	Diagnostics (Basel)	Discussed advances in dual-energy CT for functional pulmonary imaging, underlining the importance of functional assessment alongside anatomical visualization
[22]	2018	Moore et al.	Cardiovascular Diagnosis and Therapy	Provided an update on the imaging techniques for acute pulmonary embolism, emphasizing the role of MIP and VR in providing detailed vascular maps and structural relationships

These studies consistently demonstrate that advanced post-processing techniques in CTPA, such as MIP, MinIP, and VR, significantly improve the visualization and diagnostic accuracy of PTE. These techniques enhance the ability to detect and characterize thrombi, assess the extent of embolic disease, and facilitate the identification of low-density structures, which is crucial in diagnosing PTE. The advancements have also allowed for reduced radiation doses without compromising image quality, which is critical for patient safety. These findings support the continuous evolution and implementation of advanced imaging technologies in clinical settings for better patient outcomes.

Limitations

The study has several limitations, including a relatively small sample size of 50 patients, which may affect the validity and generalizability of the findings. As a single-center study conducted exclusively at Saveetha Medical College and Hospital, the results may not fully apply to other institutions or populations with different healthcare practices and equipment. Selection bias may be present due to the specific inclusion criteria, such as clinical suspicion of PTE and elevated D-dimer levels, and the exclusion of patients unable to undergo contrast-enhanced imaging. Additionally, the study focused solely on CTPA without comparing it to other imaging modalities like MRI or ultrasound, which could provide complementary diagnostic insights. The quality of the advanced post-processing techniques used, such as MIP, MinIP, and VR, can be subjective and heavily dependent on the operator's expertise. Furthermore, the imaging data were obtained over a specific period, potentially overlooking recent technological advancements that could influence diagnostic accuracy. The absence of long-term follow-up data also limits the assessment of the clinical impact of the findings on patient outcomes and treatment effectiveness.

## Conclusions

CTPA, when used alongside advanced post-processing techniques, has shown exceptional accuracy in diagnosing PE, particularly in high-risk patients. These methods enable the precise visualization of the pulmonary arteries and the detection of even small emboli, leading to more informed clinical decisions and improved patient outcomes. Given the complexities and subtlety involved in diagnosing PE, these imaging advancements are extremely valuable. The results highlight the necessity of integrating these techniques into standard practice for the prompt and accurate diagnosis of PE, thereby ensuring timely and effective treatment.
